# Forensic identification using airDNA: a preliminary study on the collection, isolation, amplification and sequencing of human DNA from air samples

**DOI:** 10.55730/1300-0144.6029

**Published:** 2025-03-03

**Authors:** Sıtkı TIPLAMAZ, Zehra ÖMEROĞLU ULU, Bestami ÇOLAK, Hızır ASLIYÜKSEK, Fikrettin ŞAHİN

**Affiliations:** 1 Department of Forensic Medicine, Faculty of Medicine, Marmara University, İstanbul, Turkiye; 2 Department of Genetics and Bioengineering, Faculty of Engineering, Yeditepe University, İstanbul, Turkiye; 3 Department of Biology, Presidency of Council of Forensic Medicine, İstanbul, Turkiye

**Keywords:** eDNA, mtDNA, next generation sequencing, likelihood ratio

## Abstract

**Background/aim:**

Humans continuously release skin cells and DNA fragments into the air. This DNA can either remain airborne or settle onto surfaces as indoor dust. This study investigates the collection, isolation, amplification, and sequencing of human DNA from air samples, focusing on both nuclear and mitochondrial DNAs.

**Materials and methods:**

This study was conducted in a 15 m^3^ room and a meeting room, where air samples were systematically collected and filtered using a vacuum device equipped with various sterile filters. Rigorous protocols were implemented to prevent contamination throughout the process. Thirteen air samples were processed in this research, involving the participation of two females and two males.

**Results:**

The study demonstrated the feasibility of extracting and sequencing human mtDNA from air samples, while nuclear DNA quantification remained challenging. Notably, cotton filters yielded better mtDNA sequencing results compared to glass fiber filters. Despite limited nuclear DNA recovery, mtDNA sequencing was successful in several samples, providing interpretable DNA profiles.

**Conclusion:**

This preliminary study highlights the potential use of airDNA in forensic investigations, emphasizing mtDNA sequencing as a viable approach when nuclear DNA is scarce. Future studies should prioritize mtDNA sequencing and consider using glass fiber or cotton filters for optimal airDNA collection.

## 1. Introduction

Environmental DNA (eDNA) is genetic material extracted from environmental samples such as soil, water, or air. eDNA is shed by organisms into their surroundings through various biological materials such as skin cells, feces, urine, hair, saliva, breath, and other bodily fluids. Today, eDNA is a powerful tool for biodiversity monitoring. The production of eDNA relies on the organism’s biomass, age, life cycle, feeding behavior, physiology, and space utilization [1]. The occurrence of human DNA in eDNA is termed as “human genetic bycatch”. Whitmore et al. demonstrated that intentionally recoverable identifiable human DNA can be obtained from human-focused environmental sampling, including air. They sequenced the nuclear DNA and utilized quantitative polymerase chain reaction (qPCR) to amplify specific gene regions in humans [2]. However, these regions are unsuitable for forensic purposes, as they do not involve short tandem repeats (STR) or single nucleotide polymorphism (SNP) regions. Physical evidence is an important tool to prove or deny criminal activity. Extracting human DNA from the air could serve as crucial evidence in shedding light on a crime. This study is a preliminary research to determine the best method to collect, isolate, amplify, and/or sequence human DNA from air. Previous studies generally reported obtaining either complex mixtures or partial/no DNA profiles from air samples, except in one instance where a full profile was obtained [3–6]. Our focus extends beyond nuclear DNA; we also sequenced mitochondrial DNA (mtDNA), given the limited yield and degraded nature of DNA in air samples.

## 2. Materials and methods

### 2.1. Preparation for sample collection

**Figure 1:**
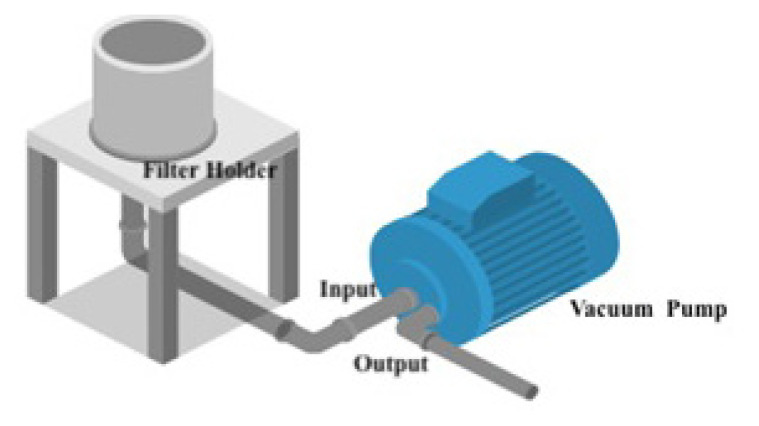
Air sampler device.

Air samples were collected from a 15 m^3^ room and a meeting room at the Marmara University Pendik Training and Research Hospital. Notably, the room lacked windows or ventilation units. The floor was cleaned using >3% bleach, and surfaces such as a vacuum pump, pipes, tables, and chairs were cleaned with >5% bleach and 70% alcohol solution, then subsequently UV-irradiated for 15 min. The meeting room was not cleaned. The filter holder was autoclaved. The person performing these tasks wore a disposable cap, mask, protective goggles, apron, shoe covers, and gloves throughout the procedure. This procedure was performed after each sampling. An oil-free vacuum pump was used with an airflow of 81–90 L/min and a maximum vacuum of 60 Torr (Kawake Airvoc Co. Ltd., Taipei City, Taiwan). Filter holders and intermediate pipes were employed in this process (Figure 1). The vacuum setup was cleaned with a mixture of >5% bleach and 70% alcohol solution after each use and left to dry. Sterile filters were carefully inserted into the filter holder using disposable forceps.

To assess contamination before participants entered the room, room air was filtered for 30 min using a vacuum device equipped with a sterile filter. This process was repeated after the cleaning and UV-irradiation of the room. The filter holder was positioned 45 cm above the floor at the center of the room. The filter was then removed using sterile forceps and transferred to a sterile petri dish. To prevent air exposure, the edges were sealed with tape before storage at −20 °C.

### 2.2. Participants

Four hospital staff members (two females and two males) were included in the study with informed consent. Before entering the room for each sampling session, they washed their hands, wiped their phones with 70% alcohol, and wore disposable overshoes to carry a minimum amount of foreign DNA from outside. They spent 1 h in the room during each sampling session. After they left the room, filtering began after 5 min and continued for 30 min for each sampling.

### 2.3. Filters

Air filtration involved the use of various filters, including a 47 mm glass fiber filter with a 45 μm pore size, a 47 mm cellulose acetate filter with a 45 μm pore size, a 50 mm disc-shaped UV-irradiated cotton filter, a cellulose acetate syringe filter with a 45 μm pore size, and a HEPA filter. Glass fiber filters were heated at 450 °C for at least 5 h to destroy any preexisting organic residues at Yeditepe University Calibration Laboratory (YUKAL) [7].

### 2.4. DNA extraction

**Figure 2:**
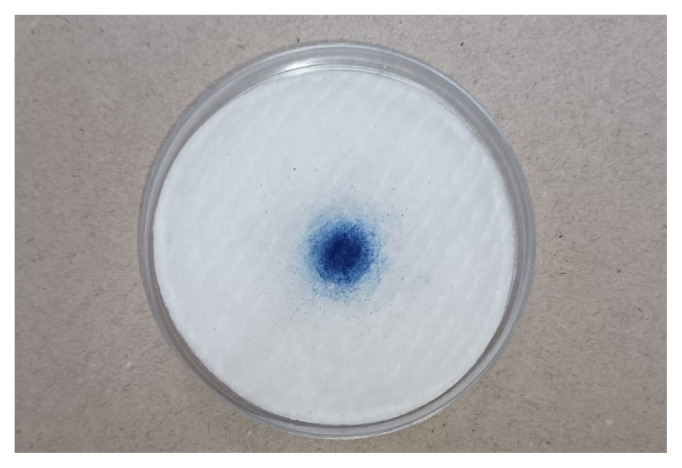
A 50 mm cotton filter in a petri dish, after sampling blue smoke.

After completing all the air samplings for genetic analysis, a blue smoke flare was ignited in the room to determine the sampling localization of the filter. Air was subsequently filtered to assess the distribution of the blue smoke on the filter. The presence of the blue color on the filter indicated the sampling area (Figure 2). A 1 cm^2^ area of the filter was incised using a sterile scalpel and placed into a spin basket tube. Subsequently, 280 μL of ATL buffer and 20 μL of proteinase K were added. The mixture was incubated at 56 °C and 1200 rpm for 1 h, followed by a 3 min centrifugation at 13,300 rpm. Finally, 1 μL of carrier RNA was added to the lower tube where the samples were collected. DNA samples were extracted using the Investigator STAR Lyse and Prep Kit (Qiagen, Hilden, Germany) according to the manufacturer’s instructions on a TECAN Freedom Evo 150 workstation (Tecan Trading AG, Männedorf, Switzerland) with an elution volume of 80 μL.

### 2.5. PCR

DNA yields were quantified using the Quantifiler Trio DNA Quantification Kit (Thermo Fisher Scientific Inc., Waltham, MA, USA) on a QuantStudio 5 real-time PCR system (Thermo Fisher Scientific Inc., Waltham, MA, USA). DNA was amplified using the PowerPlex Fusion 6C System (Promega Co., Madison, WI, USA). The cycling conditions were as per the manufacturer’s recommendations for 29 cycles with 400 pg of DNA or a maximum DNA volume of 15 μL if 400 pg was not available, in a final volume of 25 μL. Amplification products were run on an Applied Biosystems 3500xL Genetic Analyzer (Thermo Fisher Scientific Inc., Waltham, MA, USA). The data were analyzed using GeneMapper ID-X v1.6 (Thermo Fisher Scientific Inc., Waltham, MA, USA), with an analytical threshold (AT) set to 50 RFU.

### 2.6. Next-generation sequencing and analysis

Following DNA extraction and quantification, mtDNA control region sequencing was carried out using the ForenSeq mtDNA Control Region Kit (Verogen Inc. San Diego, CA, USA), according to the manufacturer’s recommendations. A total of 122 primers were used to amplify the mtDNA control region efficiently. The primer mixes consist of tagged oligonucleotides corresponding to each target amplicon. During the PCR amplification cycles, these tags were incorporated into the copies of each amplicon. As a result, the target amplicons were flanked by universal primer binding sequences. Subsequently, libraries were generated from each multiplex by linking index adapters specific to each sample to the amplicon tags of each amplicon. This process involved additional PCR cycles. The resulting two multiplexes for each library were purified before being combined and prepared for sequencing. DNA samples were sequenced on the MiSeq FGx platform (Illumina Inc. San Diego, USA). Alignment and variant calling of control samples were performed using Universal Analysis Software v2.0 (Verogen Inc., San Diego, CA, USA). The use of controls (negative amplification, reagent blank, and positive controls) was maintained throughout the laboratory process.

mtDNA is inherited as a haplotype block; therefore, Hardy–Weinberg equilibrium cannot be applied to mtDNA analysis [8,9]. The likelihood ratios (LR) for each of the questioned samples (Q1, Q2, Q3, and Q4) were calculated based on their matched mtDNA SNP blocks with the known samples (S26, T7, T8) using the EMPOP database. The LRs of STRs were calculated according to STR frequencies obtained from the STRIDER database.

## 3. Results

A total of thirteen air samples were processed. No detectable DNA was quantified in the filters obtained from the cleaned room. DNA was, however, identified on the glass fiber and cotton filters, while the remaining filters showed no DNA presence. The sample size is too small to determine which filter was statistically significantly superior (p > 0.05), but nuclear DNA was quantified in two of the cotton filters compared to only one in the glass fiber filter, despite the glass fiber filter having a larger sample size. Additionally, mtDNA sequencing read counts and detected STR peak counts were higher in the cotton filter than in the glass fiber filter.

We attempted to profile STRs from three filters (S7, S8, and T7) in which nuclear DNA had been quantified and obtained mixed partial STR profiles. The capillary electrophoresis results revealed a range of zero to five peaks in the STR regions. Additionally, we sought to quantify the nuclear DNA and sequence the mtDNA control region from the four filters (S26, S30, T7, and T8), but we could only quantify nuclear DNA from one sample. However, we could sequence mtDNA from three of these four filters. The overall results are summarized in Table 1. The STR results are shown in Table 2 and mtDNA sequencing results are shared in Table 3. Further details, including STR profiles, mtDNA sequences, and read counts, are provided in the supplementary data.

Hardy–Weinberg equilibrium cannot be applied to mtDNA analysis because mtDNA is inherited as a haplotype block [8,9]. The calculated LRs for each of the questioned samples (Q1, Q2, Q3, and Q4) based on their matched mtDNA SNP blocks with the known samples (S26, T7, T8) using the EMPOP database are shared in Table 4. EMPOP queries are shared in the supplementary data. Higher values indicate stronger evidence supporting the match. The calculated LRs from STRs and STR frequencies obtained from the STRIDER database are provided in the supplementary data.

## 4. Discussion

The extraction of eDNA from air remains a relatively unexplored area, with limited understanding of the factors influencing the process. In 2021, Clare et al. demonstrated that animal DNA can be extracted directly from air under highly controlled laboratory conditions [10]. The same scientific team and another group conducted similar research, extracting eDNA from air in a zoo and identifying exotic vertebrate species using that eDNA. Although both teams hypothesized the same, they used different filtration methods [11,12]. The duration of filtration and airflow parameters were decided with the help of data from those studies. They targeted mtDNA regions—16S and COI—to differentiate the species, not the D-loop region, which is used for forensic identification. In another study, the collection and transfer of DNA within a working forensic exhibit storage area was examined, and nuclear DNAs of both staff members and unidentified people were found on shelves [13]. However, the first forensic-purpose study, published in 2022, revealed that human DNA can be collected from the air. The study provided some of the STR genotypes of individuals (just one person with a full profile) who had recently spent time in a room. In the case of rooms that were not recently occupied, the use of dust samples instead of air samples was recommended in that study [3]. The same research team published a recent study and used the same method, but with more samples. They collected 40 air and 144 dust samples. The DNA quantities in air samples were notably lower (median = 0.1 ng) than those in dust samples (median = 2.3 ng). Among the air samples, 7.5% (N = 3) did not yield any DNA profile, 27.5% (N = 11) resulted in partial single-source profiles, and 65% (N = 26) exhibited DNA mixtures with varying numbers of detected alleles [4]. Their study differed from ours as they used a commercial air filtering device and filtered the air for a 2 h period (30 min in this study), without sequencing the mtDNA. Another research group also examined air as a potential source of forensic identification. Their study was more structured than the previous ones but yielded similar results [5]. They did not investigate the airDNA in a controlled group and also did not target the mtDNA. Within a mitochondrion, there exist two to 10 copies of mitochondrial DNA (mtDNA), and an individual somatic cell has the potential to harbor as many as 1000 mitochondria or more. mtDNA sequencing is routinely used in forensic science, especially in cases with degraded DNA (such as burnt or long-deceased bodies) for identification purposes. Therefore, in scenarios like airDNA where the available DNA is limited in quantity or has undergone degradation, opting for mtDNA sequencing enhances the probability of achieving a successful DNA typing outcome compared to targeting STR regions within nuclear DNA. The control region of mtDNA is the largest noncoding portion, yet it remains one of the most discriminatory single genetic markers for forensic identification [14]. However, there is a limitation to using mtDNA-unlike nuclear DNA, mtDNA is exclusively inherited from the mother. In cases where the separation of individuals from the same maternal lineage is necessary, mtDNA sequencing should be complemented with additional methods, such as SNPs on nuclear DNA. Despite the challenge of quantifying nuclear DNA on three out of four filters, we successfully sequenced mtDNA on three of the four filters, providing interpretable DNA profiles and LR results. Another challenge of using mtDNA is deconvolution and interpretation of results, especially in mixed mtDNA results. While STR profiling of an airDNA sample may display two alleles in each region, falsely making it appear to be from a single source, sequencing the mtDNA control region provides a clearer distinction, facilitating the determination of whether the sample originated from a single source or multiple sources. Although mtDNA sequencing is not an optimal choice for precise forensic identification, it can be used as an exclusion tool, which is crucial for solving forensic cases.

AirDNA is typically a mixed and trace source and can be easily contaminated by foreign DNA. As observed in this study, we detected foreign STR fragments and nonmatched mtDNA sequences in our samples. The recovery of air from a crime scene is not only considered evidence but also proves valuable in identifying airborne contamination of other biological samples and could be used as a control tool when contamination is suspected. The complex mixed mtDNA sequencing results do not provide a clear pattern for deconvolution and interpretation. Although these drawbacks have not been fully resolved, bioinformatic experts have attempted to address them and have recommended tools for this purpose [15–19].

This preliminary study demonstrates the potential of airDNA for forensic identification, particularly through the successful sequencing of mtDNA, even though nuclear DNA could not be quantified. While challenges remain in extracting DNA and mitigating contamination risks, the findings lay the groundwork for future advancements in airDNA technology. Sequencing mtDNA from air helps address the challenge of limited-yield and degraded nuclear DNA—often resulting in partial or no profiles—and provides haplotypes that guide us in determining investigative directions. By addressing the current limitations and expanding research efforts, airDNA could become a valuable tool in the forensic scientist’s toolkit, offering new insights and evidence in complex investigations. Future efforts should focus on increasing the number of samples studied for validation, improving DNA recovery by adjusting the filtration process (for instance, by modifying filtration duration or vacuum pressure), and developing robust software tools to deconvolute and interpret mixed nuclear and mitochondrial DNA results.

## Supplementary Information



## Figures and Tables

**Table 1:** The summary of the results.

Sample code	Filter type	Location	Participant count	DNA quantification	NGS-mtDNA
**S1**	HEPA	Room	0	0	x
**S2**	Cellulose	Room	0	0	x
**S3**	Glass fiber	Room	0	0	x
**S4**	Cotton	Room	0	0	x
**S5**	HEPA	Room	0	0	x
**S6**	Cellulose	Room	4	0	x
**S7**	Glass Fiber	Room	4	0.001 ng/μL	x
**S8**	Cotton	Room	4	0.001 ng/μL	x
**S9**	Syringe-cellulose	Room	4	0	x
**S26**	Glass fiber	Room	2	0	Mixed mtDNA
**S30**	Glass fiber	Room	4	0	0
**T7**	Cotton	Meeting room	4 (not sterilized before sampling)	0.0014 ng/μL	Mixed mtDNA
**T8**	Glass fiber	Meeting room	4 (not sterilized before sampling)	0	Mixed mtDNA

**Note:** “x”, not run.

**Table 2:**
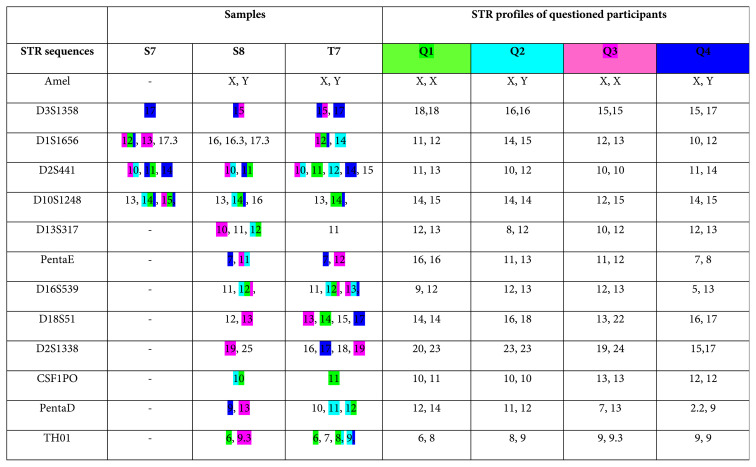

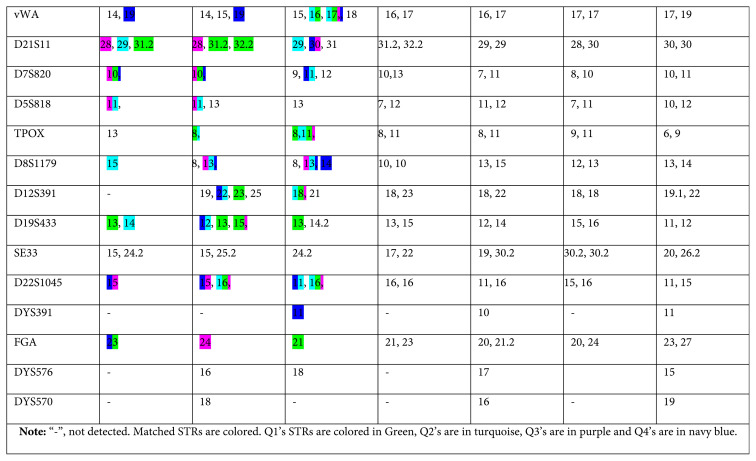
Detected STRs on sample and STR profiles of the participants.

**Table 3:** Matched mtDNA sequence results.

Samples/questioned people	Q1 (has 12 specific regions)	Q2 (has 15 specific regions)	Q3 (has 13 specific regions)	Q4 (has 10 specific regions)
S26	73G, 263G, 315.1C, 523a, 524c, 16183c, 16189C **(Match ratio: 7/12)**	73G, 189G, 194T, 195C, 204C, 263G, 309.1C, 315.1C, 16192T, 16223T, 16519C **(Match ratio: 11/15)**	x	x
73G, 152 Y, 189R, 194Y,195Y, 204Y, 210G, 263G,309.1C, 309.2C, 315.1C, 391C, 462Y,489Y, 523a, 524c,16140C, 16182M, 16183M, 16189Y, 16192Y, 16223T, 16266M, 16519C				
T7	73G, 263G, 315.1C, 523a, 524c **(Match ratio: 5/12)**	73G, 189G, 195C, 204C, 263G, 309.1C, 315.1C, 16192T, 16223T, 16292T, 16519C **(Match ratio: 11/15)**	263G, 309.1C, 315.1C, 16223Y **(Match ratio: 4/13)**	73G, 150T, 195C, 263G, 315.1C, 523a, 524c, 16223T, 16519C **(Match ratio: 9/10)**
73R, 93R, 150Y, 152Y, 189R, 195Y, 204Y, 249a, 263G, 309.1c, 315.1C, 462Y, 489Y, 497Y, 523a, 524c, 534Y, 16126Y, 16192Y, 16223Y, 16245Y, 16292Y, 16294Y, 16311Y, 16362Y, 16519Y				
T8	73G, 263G, 315.1C, 523a, **(Match ratio: 4/12)**	73G, 263G, 309.1c, 315.1C, 16519C **(Match ratio: 5/15)**	263G, 309.1C, 315.1C **(Match ratio: 3/13)**	73G, 150T, 263G, 315.1C, 523a, 16519C **(Match ratio: 6/10)**
73R, 93R, 150Y, 152Y, 263G, 309.1c, 310Y, 315.1c, 340Y, 497Y, 523a, 16070R, 16224Y, 16311Y, 16365Y, 16519Y				

**Note:** “x”, not run. Extended IUPAC notation was used for mtDNA. Match ratio: Number of matched SNPs detected in air sample/ Number of SNPs in questioned person buccal sample.

**Table 4:** Likelihood ratios calculated according to mtDNA sequencing results.

Compared samples (the questioned person sample vs the filtered air sample)	Likelihood ratio	Likelihood ratio of Σx^2^
Q1 vs S26	7.4025	5.48×10
Q2 vs S26	9.8951×10^2^	7.3274×10^5^
Q1 vs T7	1.2957	1.6788
Q2 vs T7	9.4233×10^2^	8.8799×10^5^
Q3 vs T7	1.811	3.2797
Q4 vs T7	4.7179×10^1^	2.2259×10^3^
Q1 vs T8	1.2957	1.6788
Q2 vs T8	2.0763	4.311
Q3 vs T8	1.8106	3.2783
Q4 vs T8	1.59×10^1^	2.4336×10^2^

Note: “Σx^2^”: The probability of two randomly selected individuals from a population having identical mtDNA types.
