# Supplementary material for: Forensic identification using airDNA: a preliminary study on the collection, isolation, amplification and sequencing of human DNA from air samples
Source: Turk J Med Sci. 2025 Mar 3;55(3):802–9. doi: 10.55730/1300-0144.6029 (PMC12270289; doi:10.55730/1300-0144.6029)
Supplement: Supplementary file 1 [file EMPOP_Q1S26.pdf]

EMPOP mtDNA database

**Sample ID**	Q1 in S26
**Ranges**	73 263 315.1 523 524 16183 16189
**Profile**	73G 263G 315.1C 523a 524c 16183c 16189C

alignPhyloEmp v1.15retro 27.10.2021
alignPhyloFst v1.15retro 27.10.2021
searchCostEmp v1.14retro 27.10.2021
searchCostFst v1.14retro 27.10.2021
searchCountEmp v1.14retro 27.10.2021
searchCountFst v1.14retro 27.10.2021

Origin		Frequency	Clopper Pearson CI	(x + 1)/(n + 1)
Europe	380/8321	4.5668e-2	[4.1282e-2, 5.0375e-2]	4.5782e-2
Asia	1793/10954	1.6368e-1	[1.5680e-1, 1.7075e-1]	1.6376e-1
America	2865/18113	1.5817e-1	[1.5289e-1, 1.6357e-1]	1.5822e-1
Africa	257/2378	1.0807e-1	[9.5874e-2, 1.2125e-1]	1.0845e-1
Oceania	89/96	9.2708e-1	[8.5552e-1, 9.7018e-1]	9.2784e-1

Metapopulation		Frequency	Clopper Pearson CI	(x + 1)/(n + 1)
Sub-Saharan African	572/5351	1.0690e-1	[9.8740e-2, 1.1548e-1]	1.0706e-1
Westeurasian	969/16232	5.9697e-2	[5.6100e-2, 6.3452e-2]	5.9755e-2
South Asian	88/1280	6.8750e-2	[5.5501e-2, 8.4018e-2]	6.9477e-2
East Asian	972/4180	2.3254e-1	[2.1980e-1, 2.4565e-1]	2.3272e-1
Southeast Asian	756/2994	2.5251e-1	[2.3702e-1, 2.6847e-1]	2.5275e-1
Native American	1622/7455	2.1757e-1	[2.0825e-1, 2.2711e-1]	2.1768e-1
Admixed	316/2274	1.3896e-1	[1.2500e-1, 1.5386e-1]	1.3934e-1
Oceania	89/96	9.2708e-1	[8.5552e-1, 9.7018e-1]	9.2784e-1
