# Supplementary material for: Forensic identification using airDNA: a preliminary study on the collection, isolation, amplification and sequencing of human DNA from air samples
Source: Turk J Med Sci. 2025 Mar 3;55(3):802–9. doi: 10.55730/1300-0144.6029 (PMC12270289; doi:10.55730/1300-0144.6029)
Supplement: Supplementary file 3 [file EMPOP_Q1T8.pdf]

EMPOP mtDNA database

**Sample ID**	Q1 in T8
**Ranges**	73 263 315.1 523
**Profile**	73G 263G 315.1C 523a

alignPhyloEmp v1.15retro 27.10.2021
alignPhyloFst v1.15retro 27.10.2021
searchCostEmp v1.14retro 27.10.2021
searchCostFst v1.14retro 27.10.2021
searchCountEmp v1.14retro 27.10.2021
searchCountFst v1.14retro 27.10.2021

Origin		Frequency	Clopper Pearson CI	(x + 1)/(n + 1)
Europe	4589/8321	5.5150e-1	[5.4073e-1, 5.6222e-1]	5.5155e-1
Asia	9565/10954	8.7320e-1	[8.6682e-1, 8.7937e-1]	8.7321e-1
America	14711/18113	8.1218e-1	[8.0641e-1, 8.1784e-1]	8.1219e-1
Africa	1804/2378	7.5862e-1	[7.4090e-1, 7.7570e-1]	7.5872e-1
Oceania	96/96	1.0000e+0	[9.6230e-1, 1.0000e+0]	1.0000e+0

Metapopulation		Frequency	Clopper Pearson CI	(x + 1)/(n + 1)
Sub-Saharan African	4682/5351	8.7498e-1	[8.6582e-1, 8.8373e-1]	8.7500e-1
Westeurasian	9684/16232	5.9660e-1	[5.8900e-1, 6.0416e-1]	5.9662e-1
South Asian	1036/1280	8.0937e-1	[7.8677e-1, 8.3055e-1]	8.0952e-1
East Asian	4055/4180	9.7010e-1	[9.6447e-1, 9.7505e-1]	9.7010e-1
Southeast Asian	2954/2994	9.8664e-1	[9.8185e-1, 9.9044e-1]	9.8664e-1
Native American	6503/7455	8.7230e-1	[8.6451e-1, 8.7980e-1]	8.7232e-1
Admixed	1755/2274	7.7177e-1	[7.5395e-1, 7.8888e-1]	7.7187e-1
Oceania	96/96	1.0000e+0	[9.6230e-1, 1.0000e+0]	1.0000e+0
