# Supplementary material for: Forensic identification using airDNA: a preliminary study on the collection, isolation, amplification and sequencing of human DNA from air samples
Source: Turk J Med Sci. 2025 Mar 3;55(3):802–9. doi: 10.55730/1300-0144.6029 (PMC12270289; doi:10.55730/1300-0144.6029)
Supplement: Supplementary file 4 [file EMPOP_Q2S26.pdf]

EMPOP mtDNA database

**Sample ID**	Q2 in S26
**Ranges**	73 189 194 195 204 263 309.1 315.1 16192 16223 16519
**Profile**	73G 189G 194T 195C 204C 263G 309.1C 315.1C 16192T 16223T 16519C

alignPhyloEmp v1.15retro 27.10.2021
alignPhyloFst v1.15retro 27.10.2021
searchCostEmp v1.14retro 27.10.2021
searchCostFst v1.14retro 27.10.2021
searchCountEmp v1.14retro 27.10.2021
searchCountFst v1.14retro 27.10.2021

Origin		Frequency	Clopper Pearson CI	(x + 1)/(n + 1)
Europe	12/8228	1.4584e-3	[7.5381e-4, 2.5462e-3]	1.5798e-3
Asia	20/10815	1.8493e-3	[1.1299e-3, 2.8546e-3]	1.9416e-3
America	6/18061	3.3221e-4	[1.2192e-4, 7.2293e-4]	3.8755e-4
Africa	1/2378	4.2052e-4	[1.0647e-5, 2.3407e-3]	8.4069e-4
Oceania	0/96	0.0000e+0	[0.0000e+0, 3.7697e-2]	1.0309e-2

Metapopulation		Frequency	Clopper Pearson CI	(x + 1)/(n + 1)
Sub-Saharan African	0/5343	0.0000e+0	[0.0000e+0, 6.9018e-4]	1.8713e-4
Westeurasian	25/15971	1.5653e-3	[1.0133e-3, 2.3099e-3]	1.6278e-3
South Asian	10/1309	7.6394e-3	[3.6693e-3, 1.4004e-2]	8.3969e-3
East Asian	0/4180	0.0000e+0	[0.0000e+0, 8.8212e-4]	2.3918e-4
Southeast Asian	0/2994	0.0000e+0	[0.0000e+0, 1.2313e-3]	3.3389e-4
Native American	3/7496	4.0021e-4	[8.2541e-5, 1.1691e-3]	5.3355e-4
Admixed	1/2189	4.5683e-4	[1.1566e-5, 2.5426e-3]	9.1324e-4
Oceania	0/96	0.0000e+0	[0.0000e+0, 3.7697e-2]	1.0309e-2
