# Supplementary material for: Forensic identification using airDNA: a preliminary study on the collection, isolation, amplification and sequencing of human DNA from air samples
Source: Turk J Med Sci. 2025 Mar 3;55(3):802–9. doi: 10.55730/1300-0144.6029 (PMC12270289; doi:10.55730/1300-0144.6029)
Supplement: Supplementary file 6 [file EMPOP_Q2T8.pdf]

EMPOP mtDNA database

**Sample ID**	Q2 in T8
**Ranges**	73 263 309.1 315.1 16519
**Profile**	73G 263G 309.1c 315.1C 16519C

alignPhyloEmp v1.15retro 27.10.2021
alignPhyloFst v1.15retro 27.10.2021
searchCostEmp v1.14retro 27.10.2021
searchCostFst v1.14retro 27.10.2021
searchCountEmp v1.14retro 27.10.2021
searchCountFst v1.14retro 27.10.2021

Origin		Frequency	Clopper Pearson CI	(x + 1)/(n + 1)
Europe	3089/8228	3.7543e-1	[3.6495e-1, 3.8599e-1]	3.7550e-1
Asia	6114/10815	5.6533e-1	[5.5592e-1, 5.7470e-1]	5.6537e-1
America	8584/18061	4.7528e-1	[4.6797e-1, 4.8259e-1]	4.7531e-1
Africa	1184/2378	4.9790e-1	[4.7760e-1, 5.1820e-1]	4.9811e-1
Oceania	90/96	9.3750e-1	[8.6891e-1, 9.7672e-1]	9.3814e-1

Metapopulation		Frequency	Clopper Pearson CI	(x + 1)/(n + 1)
Sub-Saharan African	3057/5343	5.7215e-1	[5.5875e-1, 5.8547e-1]	5.7223e-1
Westeurasian	6449/15971	4.0379e-1	[3.9617e-1, 4.1145e-1]	4.0383e-1
South Asian	797/1309	6.0886e-1	[5.8182e-1, 6.3541e-1]	6.0916e-1
East Asian	2209/4180	5.2847e-1	[5.1320e-1, 5.4370e-1]	5.2858e-1
Southeast Asian	2097/2994	7.0040e-1	[6.8364e-1, 7.1677e-1]	7.0050e-1
Native American	3361/7496	4.4837e-1	[4.3707e-1, 4.5972e-1]	4.4845e-1
Admixed	1001/2189	4.5729e-1	[4.3626e-1, 4.7843e-1]	4.5753e-1
Oceania	90/96	9.3750e-1	[8.6891e-1, 9.7672e-1]	9.3814e-1
