# Supplementary material for: Forensic identification using airDNA: a preliminary study on the collection, isolation, amplification and sequencing of human DNA from air samples
Source: Turk J Med Sci. 2025 Mar 3;55(3):802–9. doi: 10.55730/1300-0144.6029 (PMC12270289; doi:10.55730/1300-0144.6029)
Supplement: Supplementary file 8 [file EMPOP_Q3T8.pdf]

EMPOP mtDNA database

Sample ID Q3 in T8
Ranges 263 309.1 315.1
Profile 263G 309.1C 315.1C

---

alignPhyloEmp v1.15retro 27.10.2021
alignPhyloFst v1.15retro 27.10.2021
searchCostEmp v1.14retro 27.10.2021
searchCostFst v1.14retro 27.10.2021
searchCountEmp v1.14retro 27.10.2021
searchCountFst v1.14retro 27.10.2021

Origin		Frequency	Clopper Pearson CI	(x + 1)/(n + 1)
Europe	6834/12795	5.3411e-1	[5.2543e-1, 5.4279e-1]	5.3415e-1
Asia	7068/12149	5.8178e-1	[5.7295e-1, 5.9057e-1]	5.8181e-1
America	10742/19081	5.6297e-1	[5.5590e-1, 5.7002e-1]	5.6299e-1
Africa	1093/2577	4.2414e-1	[4.0496e-1, 4.4349e-1]	4.2436e-1
Australia (Continent)	153/305	5.0164e-1	[4.4411e-1, 5.5914e-1]	5.0327e-1
Oceania	70/96	7.2917e-1	[6.2886e-1, 8.1485e-1]	7.3196e-1

Metapopulation		Frequency	Clopper Pearson CI	(x + 1)/(n + 1)
Sub-Saharan African	2297/5437	4.2248e-1	[4.0930e-1, 4.3574e-1]	4.2258e-1
Westeurasian	11645/21514	5.4128e-1	[5.3459e-1, 5.4795e-1]	5.4130e-1
South Asian	867/1540	5.6299e-1	[5.3779e-1, 5.8794e-1]	5.6327e-1
East Asian	2740/4735	5.7867e-1	[5.6445e-1, 5.9279e-1]	5.7876e-1
Southeast Asian	1865/2996	6.2250e-1	[6.0486e-1, 6.3990e-1]	6.2262e-1
Native American	4807/7727	6.2210e-1	[6.1118e-1, 6.3293e-1]	6.2215e-1
Admixed	1668/2955	5.6447e-1	[5.4636e-1, 5.8244e-1]	5.6461e-1
Oceania	71/99	7.1717e-1	[6.1778e-1, 8.0314e-1]	7.2000e-1
