# Supplementary material for: Forensic identification using airDNA: a preliminary study on the collection, isolation, amplification and sequencing of human DNA from air samples
Source: Turk J Med Sci. 2025 Mar 3;55(3):802–9. doi: 10.55730/1300-0144.6029 (PMC12270289; doi:10.55730/1300-0144.6029)
Supplement: Supplementary file 9 [file EMPOP_Q4T7.pdf]

Sample ID Q4 in T7
Ranges 73 150 195 263 315.1 523 524 16223 16519
Profile 73G 150T 195C 263G 315.1C 523a 524c 16223T 16519C

alignPhyloEmp v1.15retro 27.10.2021
alignPhyloFst v1.15retro 27.10.2021
searchCostEmp v1.14retro 27.10.2021
searchCostFst v1.14retro 27.10.2021
searchCountEmp v1.14retro 27.10.2021
searchCountFst v1.14retro 27.10.2021

Origin		Frequency	Clopper Pearson CI	(x + 1)/(n + 1)
Europe	47/8173	5.7506e-3	[4.2283e-3, 7.6399e-3]	5.8723e-3
Asia	59/10786	5.4701e-3	[4.1666e-3, 7.0504e-3]	5.5623e-3
America	588/18008	3.2652e-2	[3.0104e-2, 3.5352e-2]	3.2706e-2
Africa	141/2378	5.9294e-2	[5.0139e-2, 6.9552e-2]	5.9689e-2
Oceania	0/96	0.0000e+0	[0.0000e+0, 3.7697e-2]	1.0309e-2

Metapopulation		Frequency	Clopper Pearson CI	(x + 1)/(n + 1)
Sub-Saharan African	606/5343	1.1342e-1	[1.0504e-1, 1.2223e-1]	1.1359e-1
Westeurasian	51/15916	3.2043e-3	[2.3867e-3, 4.2110e-3]	3.2669e-3
South Asian	3/1280	2.3437e-3	[4.8360e-4, 6.8340e-3]	3.1226e-3
East Asian	18/4180	4.3062e-3	[2.5541e-3, 6.7972e-3]	4.5444e-3
Southeast Asian	30/2994	1.0020e-2	[6.7704e-3, 1.4274e-2]	1.0351e-2
Native American	47/7443	6.3147e-3	[4.6434e-3, 8.3884e-3]	6.4481e-3
Admixed	80/2189	3.6546e-2	[2.9083e-2, 4.5281e-2]	3.6986e-2
Oceania	0/96	0.0000e+0	[0.0000e+0, 3.7697e-2]	1.0309e-2
