# Supplementary material for: Forensic identification using airDNA: a preliminary study on the collection, isolation, amplification and sequencing of human DNA from air samples
Source: Turk J Med Sci. 2025 Mar 3;55(3):802–9. doi: 10.55730/1300-0144.6029 (PMC12270289; doi:10.55730/1300-0144.6029)
Supplement: Supplementary file 10 [file EMPOP_Q4T8.pdf]

EMPOP mtDNA database

**Sample ID**	Q4 in T8
**Ranges**	73 150 263 315.1 523 16519
**Profile**	73G 150T 263G 315.1C 523a 16519C

alignPhyloEmp v1.15retro 27.10.2021
alignPhyloFst v1.15retro 27.10.2021
searchCostEmp v1.14retro 27.10.2021
searchCostFst v1.14retro 27.10.2021
searchCountEmp v1.14retro 27.10.2021
searchCountFst v1.14retro 27.10.2021

Origin		Frequency	Clopper Pearson CI	(x + 1)/(n + 1)
Europe	263/8173	3.2179e-2	[2.8460e-2, 3.6237e-2]	3.2298e-2
Asia	682/10786	6.3230e-2	[5.8709e-2, 6.7989e-2]	6.3317e-2
America	1227/18008	6.8136e-2	[6.4498e-2, 7.1915e-2]	6.8188e-2
Africa	308/2378	1.2952e-1	[1.1628e-1, 1.4368e-1]	1.2989e-1
Oceania	0/96	0.0000e+0	[0.0000e+0, 3.7697e-2]	1.0309e-2

Metapopulation		Frequency	Clopper Pearson CI	(x + 1)/(n + 1)
Sub-Saharan African	957/5343	1.7911e-1	[1.6892e-1, 1.8966e-1]	1.7927e-1
Westeurasian	607/15916	3.8138e-2	[3.5215e-2, 4.1230e-2]	3.8198e-2
South Asian	37/1280	2.8906e-2	[2.0433e-2, 3.9625e-2]	2.9664e-2
East Asian	326/4180	7.7990e-2	[7.0039e-2, 8.6538e-2]	7.8211e-2
Southeast Asian	230/2994	7.6820e-2	[6.7531e-2, 8.6946e-2]	7.7129e-2
Native American	161/7443	2.1631e-2	[1.8447e-2, 2.5197e-2]	2.1762e-2
Admixed	162/2189	7.4006e-2	[6.3388e-2, 8.5782e-2]	7.4429e-2
Oceania	0/96	0.0000e+0	[0.0000e+0, 3.7697e-2]	1.0309e-2
