# Supplementary figures and images for: Forensic identification using airDNA: a preliminary study on the collection, isolation, amplification and sequencing of human DNA from air samples
Source: Turk J Med Sci. 2025 Mar 3;55(3):802–9. doi: 10.55730/1300-0144.6029 (PMC12270289; doi:10.55730/1300-0144.6029)

| Sample File | Sample Name | Panel | SQO | SOS | SQ | SSPK | MIX | OMR | CGQ |
|-------------|-------------|-------|-----|-----|----|------|-----|-----|-----|
|-------------|-------------|-------|-----|-----|----|------|-----|-----|-----|

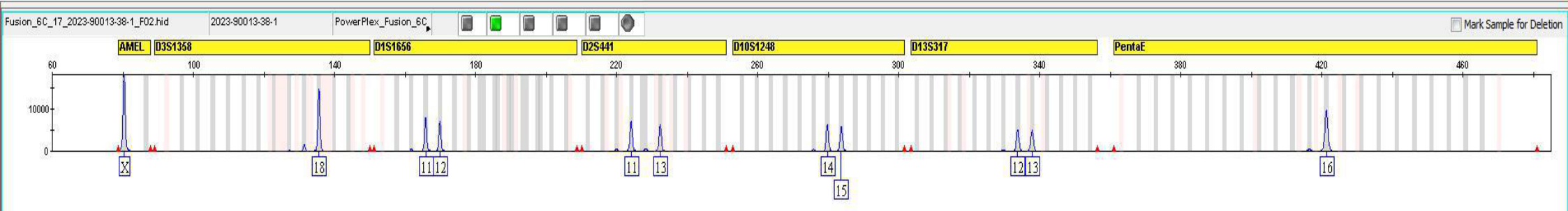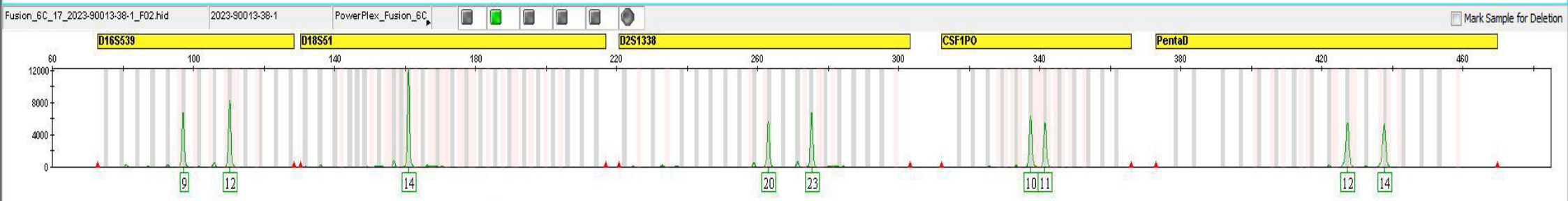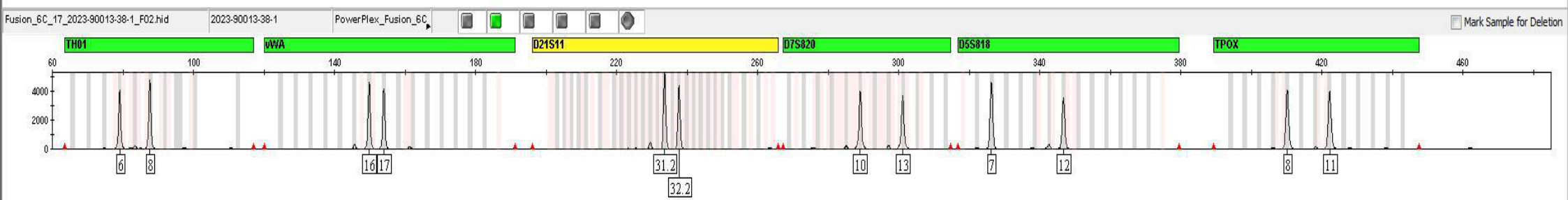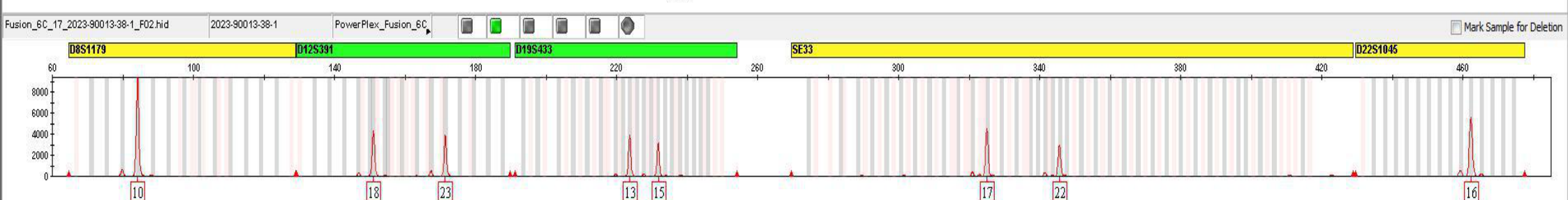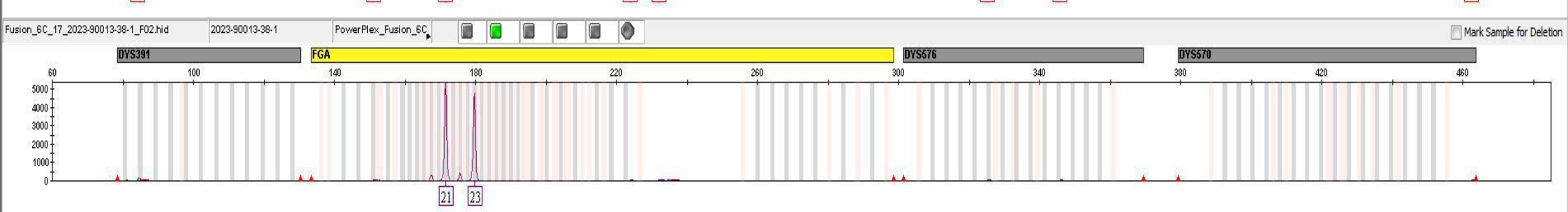

Supplement: Supplementary file 12 [file Q1STRProfiling.pdf]

| Sample File | Sample Name | Panel | SQO | SQS | SQ | SSPK | MIX | OMR | CGQ |
|-------------|-------------|-------|-----|-----|----|------|-----|-----|-----|
|-------------|-------------|-------|-----|-----|----|------|-----|-----|-----|

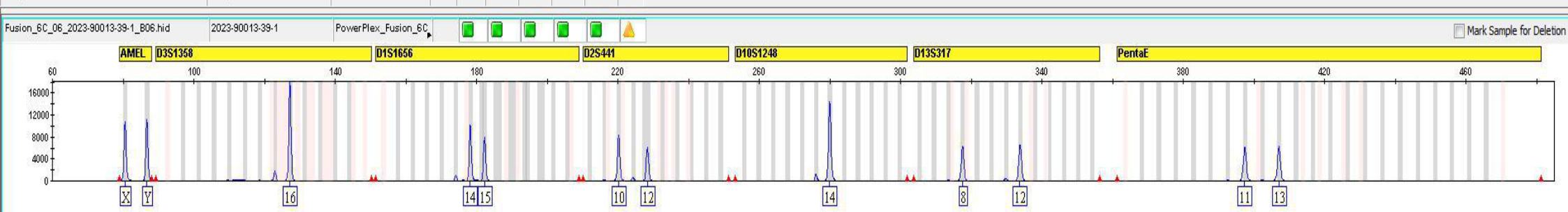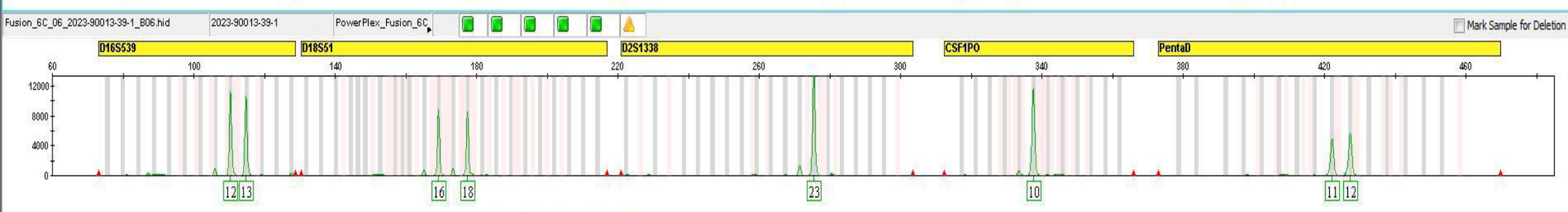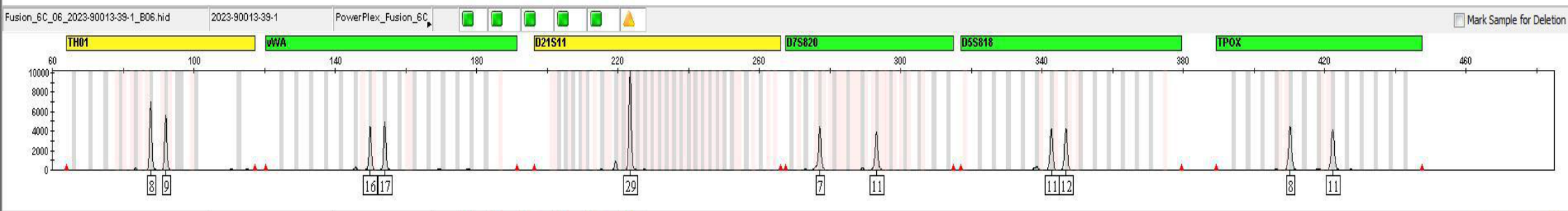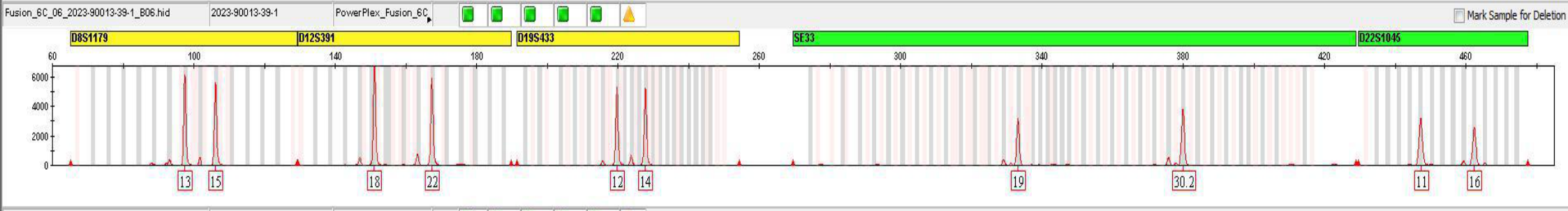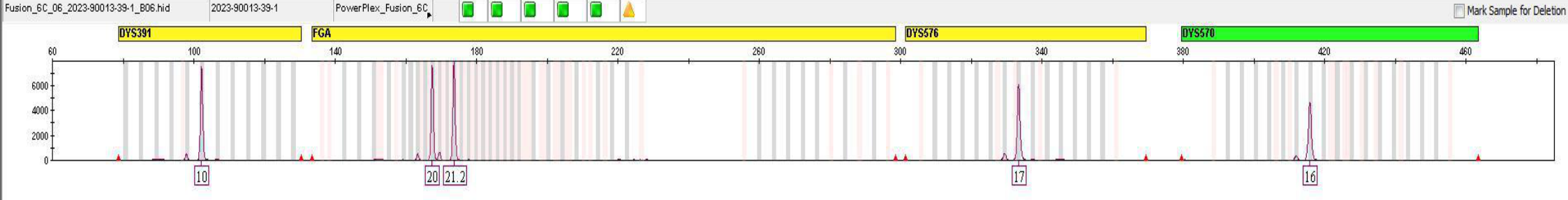

Supplement: Supplementary file 13 [file Q2STRProfiling.pdf]

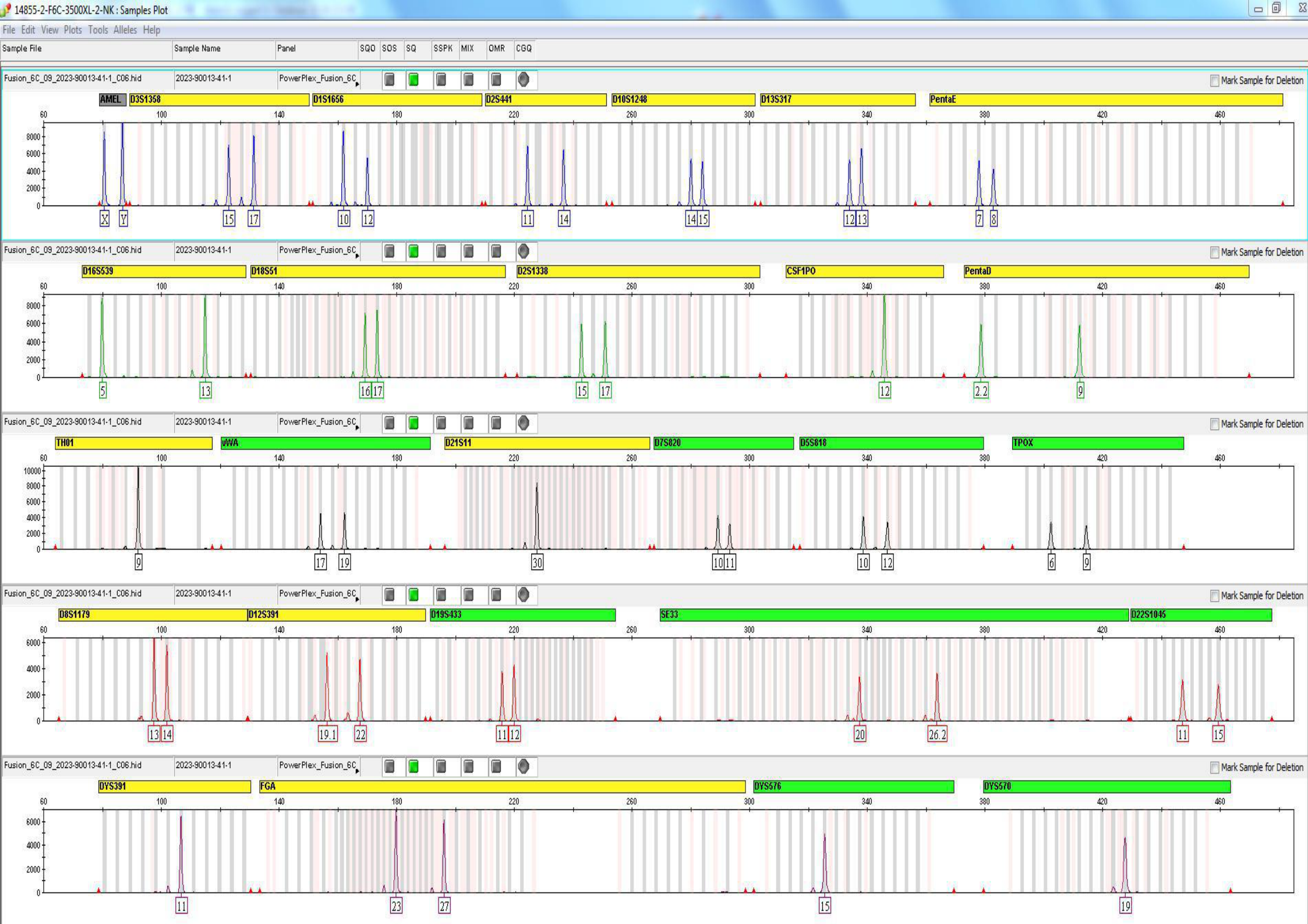

Supplement: Supplementary file 15 [file Q4STRProfiling.pdf]

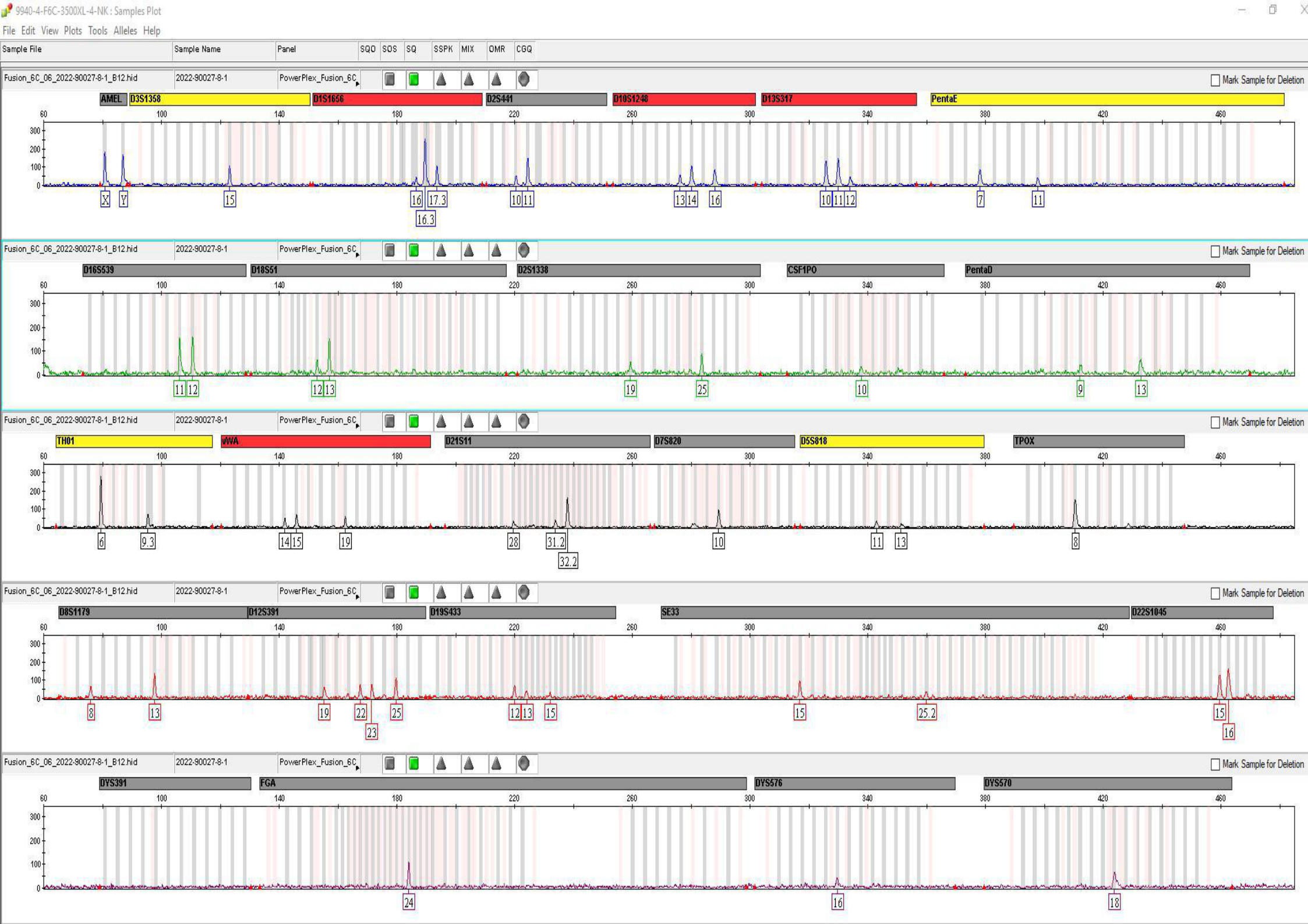

Supplement: Supplementary file 17 [file S8STRprofiling.pdf]

| Sample File | Sample Name | Panel | SQO | SOS | SQ | SSPK | MIX | QMR | CGQ |
|-------------|-------------|-------|-----|-----|----|------|-----|-----|-----|
|-------------|-------------|-------|-----|-----|----|------|-----|-----|-----|

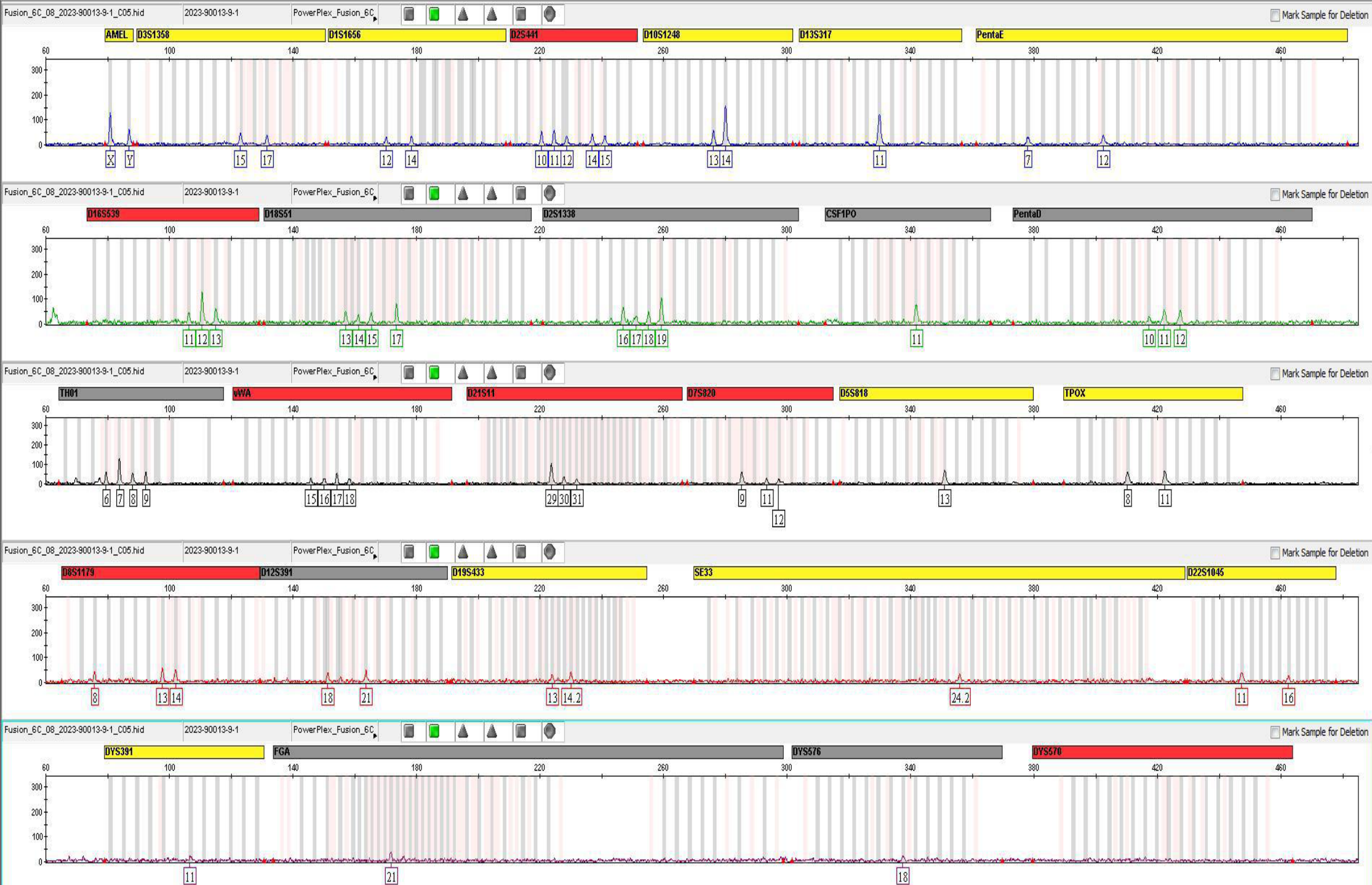

Supplement: Supplementary file 19 [file T7STRprofiling.pdf]
