# Supplementary material for: Forensic identification using airDNA: a preliminary study on the collection, isolation, amplification and sequencing of human DNA from air samples
Source: Turk J Med Sci. 2025 Mar 3;55(3):802–9. doi: 10.55730/1300-0144.6029 (PMC12270289; doi:10.55730/1300-0144.6029)

14855-4-F6C-3500XL-2-NK : Samples Plot

File Edit View Plots Tools Alleles Help

Sample File	Sample Name	Panel	SQO	SOS	SQ	SSPK	MIX	OMR	CGQ

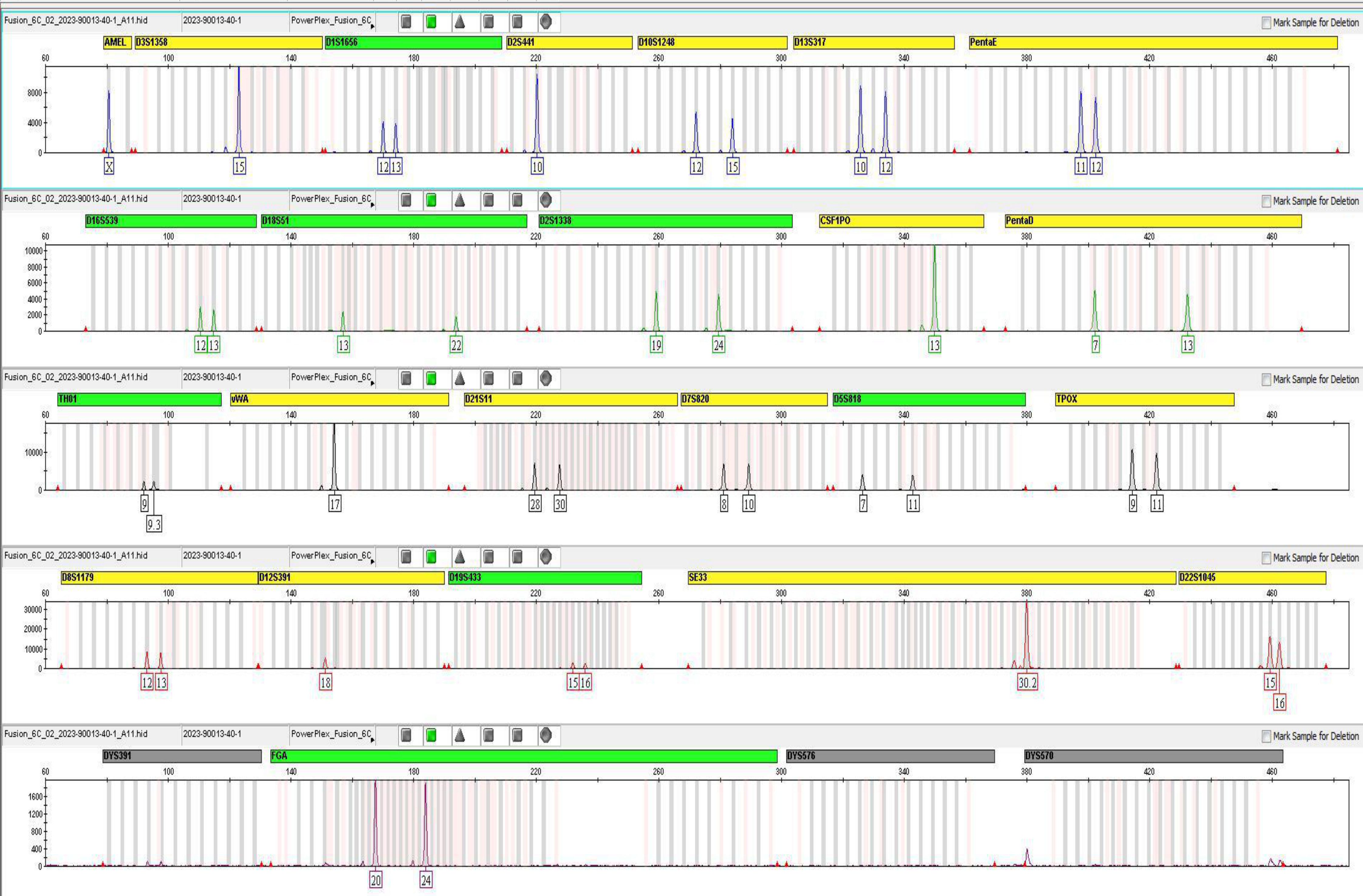

Supplement: Supplementary file 14 [file Q3STRProfiling.pdf]
