# Supplementary material for: Forensic identification using airDNA: a preliminary study on the collection, isolation, amplification and sequencing of human DNA from air samples
Source: Turk J Med Sci. 2025 Mar 3;55(3):802–9. doi: 10.55730/1300-0144.6029 (PMC12270289; doi:10.55730/1300-0144.6029)
Supplement: Supplementary file 16 [file S7STRprofiling.pdf]

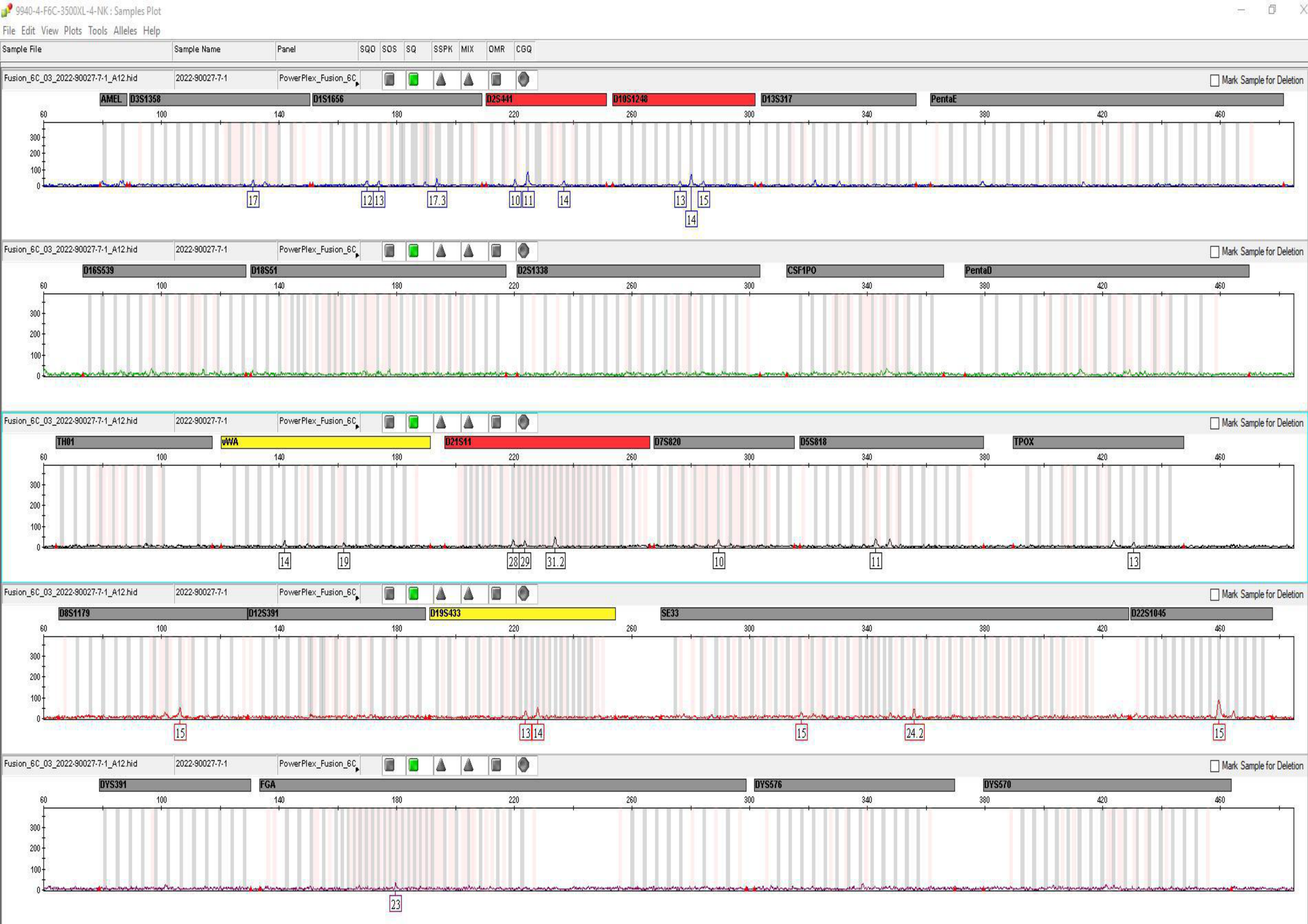
9940-4-F6C-3500XL-4-NK : Samples Plot
File Edit View Plots Tools Alleles Help
Sample File
Sample Name
Panel
SQO SOS SQ SSPK MIX OMR CGQ
Fusion_6C_03_2022-90027-7-1_A12.hid
2022-90027-7-1
PowerPlex_Fusion_6C
Mark Sample for Deletion
AMEL D3S1358 D1S1656 D2S441 D10S1248 D13S317 PentaE
17 12 13 17.3 10 11 14 13 14 15
D16S539 D18S51 D2S1338 CSF1PO PentaD
TH01 vWA D21S11 D7S820 D5S818 TPOX
14 19 28 29 31.2 10 11 13
D8S1179 D12S391 D19S433 SE33 D22S1045
15 13 14 15 24.2 15
DYS391 FGA DYS576 DYS570
23
