# Supplementary material for: Forensic identification using airDNA: a preliminary study on the collection, isolation, amplification and sequencing of human DNA from air samples
Source: Turk J Med Sci. 2025 Mar 3;55(3):802–9. doi: 10.55730/1300-0144.6029 (PMC12270289; doi:10.55730/1300-0144.6029)
Supplement: Supplementary file 18 [file STR-LRs.pdf]

STR REGIONS AND THEIR ALLEL FREQUENCIES AND CALCULATION OF THE LIKELIHOOD RATIOS

Detected questioned STR profiles (Q1, Q2, Q3, Q4) in the samples (S7, S8, T7) and Calculation of the Likelihood ratios

STRs	Q1 in S7	Q2 in S7	Q3 in S7	Q4 in S7
Amel	-	-	-	-
D3S1358		-	-	17
D1S1656	12	-	12, 13	12
D2S441	11	10	10	11, 14
D10S1248	14, 15	14	15	14, 15
D13S317	-	-	-	-
PentaE	-	-	-	-
D16S539	-	-	-	-
D18S51	-	-	-	-
D2S1338	-	-	-	-
CSF1PO	-	-	-	-
PentaD	-	-	-	-
TH01	-	-	-	-
vWA	-	-	-	19
D21S11	31.2	29	28	-
D7S820	10	-	10	10
D5S818	-	11	11,	-
TPOX	-	-	-	-
D8S1179	-	15	-	-
D12S391	-	-	-	-
D19S433	13	14	-	-
SE33	-	-	-	-
D22S1045	-	-	15	15
DYS391	-	-	-	-
FGA	23	-	-	23
DYS576	-	-	-	-
DYS570	-	-	-	-

LR of Q1 in S7

There are 7 matched str regions.

LR=1 / (0.1294 x 0.3366 x 2 x 0.3126 x 0.206 x 0.092 x 0.2621 x 0.2357 x 0.1486)= **211072.7566128601776220120374394 5**

LR of Q2 in S7

There are 6 matched str regions.

LR=1 / (0.1871 x 0.3126 x 0.2159 x 0.3359 x 0.108 x 0.3486) =**6262.149828845991938190234571053**1

LR of Q3 in S7

There are 8 matched str regions.

LR=1 / (2 x 0.1294 x 0.067 x 0.1871 x 0.206 x 0.1528 x 0.2621 x 0.3623) =**103124.34620894225833878314760478**

LR of Q4 in S7

There are 7 matched str regions.

LR=1 / (0.2105 x 0.1294 x 2 x 0.3366 x 0.2933 x 2 x 0.3126 x 0.206 x 0.0796 x 0.2621 x 0.3623 x 0.1486) = **1285298.5907111768298831009132986**

STRs	Q1 in S8	Q2 in S8	Q3 in S8	Q4 in S8
Amel	-	-	-	-
D3S1358	-	-	15	15
D1S1656	-	-	-	-
D2S441	11	10	10,	11
D10S1248	14,	14,	-	14,
D13S317	12	12	10,	-
PentaE	-	11	11	7
D16S539	12,	12,	12,	-
D18S51	-	-	13	-
D2S1338	-	-	19	-
CSF1PO	10	10	-	-
PentaD	-	-	13	9
TH01	6	-	9.3	-
vWA	-	-	-	19
D21S11	31.2, 32.2	-	28	-
D7S820	10,	-	10,	10,
D5S818	-	11	11	-
TPOX	8,	8,	-	-
D8S1179	-	13,	13,	13,
D12S391	23	22	-	22
D19S433	13, 15,	12,	15,	12
SE33	-	-	-	-
D22S1045	16,	16,	15, 16,	15
DYS391	-	-	-	-
FGA	-	-	24	-
DYS576	-	-	-	-
DYS570	-	-	-	-

LR of Q1 in S8

There are 12 matched str regions.

LR=1 / (0.3366 x 0.3126 x 0.3219 x 0.3039 x 0.259 x 0.2325 x 2 x 0.092 x 0.0991 x 0.2621 x 0.5406 x 0.0868 x 2 x 0.2357 x 0.1615 x 0.3373) = **280149470.25380659576947653393354**

LR of Q2 in S8

There are 12 matched str regions.

LR=1 / (0.1871 x 0.3126 x 0.3219 x 0.1285 x 0.3039 x 0.259 x 0.3358 x 0.5406 x 0.3248 x 0.107 x 0.0856 x 0.3373)= **28829428.48524559**

LR of Q3 in S8

There are 16 matched str regions.

LR=1 / (0.2565 x 0.3126 x 0.0613 x 0.1285 x 0.3039 x 0.1349 x 0.2621 x 0.3358 x 0.1088 x 0.2012 x 0.3054 x 0.1528 x 0.3248 x 0.1615 x 2 x 0.3623 x 0.3372 x 0.1347) = **248816052043.95984**

LR of Q4 in S8

There are 11 matched str regions.

LR=1 / (0.2565 x 0.3366 x 0.3126 x 0.1544 x 0.2082 x 0.0796 x 0.2621 x 0.3248 x 0.107 x 0.0856 x 0.3623)= **51257749.98137976**

STRs	Q1 in T7	Q2 in T7	Q3 in T7	Q4 in T7
Amel	X, Y	X, Y	X, Y	X, Y
D3S1358		-	15	15, 17
D1S1656	12,	14	12,	12,
D2S441	11,	10, 12,	10	14
D10S1248	14,	-	-	14,
D13S317	-	-	-	-
PentaE	-	-	12	7
D16S539	12,	12, 13,	12, 13,	13,
D18S51	14	-	13	17
D2S1338	-	-	19	17
CSF1PO	11	-	-	-
PentaD	12	11, 12	-	-
TH01	6, 8,	8, 9,	-	9,
vWA	16, 17,,	16, 17,,	17,,	17,,
D21S11	-	29	30	30
D7S820	-	11	-	11
D5S818	-	-	-	-
TPOX	8, 11,	8,11,	11,	-
D8S1179	-	13,	13,	13, 14
D12S391	18,	18,	18,	-
D19S433	13,	-	-	-
SE33		-	-	-
D22S1045	16,	11, 16,	16,	11
DYS391	-	-	-	11
FGA	21	-	-	-
DYS576	-	-	-	-
DYS570	-	-	-	-

LR of Q1 in T7

There are 14 matched str regions.

LR=1 / (0.1294 x 0.3366 x 0.3126 x 0.3039 x 0.1685 x 0.3193 x 0.2258 x 2 x 0.2325 x 0.1106 x 2 x 0.2074 x 0.2756 x 2 x 0.5406 x 0.2479 x 0.1855 x 0.2357 x 0.3373 x 0.0024)= **356672390846.927**

LR of Q2 in T7

There are 12 matched str regions.

LR=1 / (0.0843 x 2 x 0.1871 x 0.0392 x 2 x 0.3039 x 0.1793 x 2 x 0.149 x0.2258 x 2 x 0.1106 x 0.1739 x 2 x 0.2074 x 0.2756 x 0.2159 x 0.2039 x 2 x 0.5406 x 0.2479 x 0.3248 x 0.1855 x 2 x 0.1345 x 0.3373) = **2027814800.00000024367234961905836359170496**

LR of Q4 in T7

There are 13 matched str regions.

LR=1 / (0.2565 x 0.3366 x 0.1871 x 0.2045 x 2 x 0.3039 x 0.1793 x 0.1349 x 0.1087 x 0.2756 x 0.2398 x 0.2479 x 0.3248 x 0.1855 x 0.3373) = **67842474.236111169576544565346167933465027 9**

LR of Q4 in T7

There are 14 matched str regions.

LR=1 / (2 x 0.2565 x 0.2105 x 0.3366 x 0.2933 x 0.3126 x 0.1544 x 0.1793 x 0.1089 x 0.2261 x 0.1738 x 0.2756 x 0.2398 x 0.2039 x 2 x 0.3248 x 0.2166 x 0.1345) = **71958762.586688171261912588981966160099365 1**

The table of verbal equivalent of the likelihood ratios

Likelihood Ratio	Verbal Equivalent
**1**	is neutral
**1 – 10^1**	provides slight support
**10 – 10^2**	provides moderate support
**100 – 10^3**	provides strong support
**1000 – 10^6**	provides very strong support
**Over 10^6**	provides extremely strong support

STR frequencies obtained from the STRIDER data base (for the European population).

CSF1PO Allele 10: 0.2589575254747388

CSF1PO Allele 11: 0.319280440991928

CSF1PO Allele 12: 0.3221800738717402

CSF1PO Allele 13: 0.0561114961482939

D3S1358 Allele 15: 0.2565365471039166

D3S1358 Allele 16: 0.250387699378384

D3S1358 Allele 17: 0.210459022139703

D3S1358 Allele 18: 0.1549516053482506

D1S1656 Allele 11: 0.0836510609615921

D1S1656 Allele 12: 0.1294333805476779

D1S1656 Allele 13: 0.0669066513883336

D1S1656 Allele 14: 0.0842870096752756

D1S1656 Allele 15: 0.1359338074211121

D1S1656 Allele 16: 0.1200374941424553

D1S1656 Allele 16.3: 0.0522819280440192

D1S1656 Allele 17.3: 0.136711374801075

D2S441 Allele 10: 0.187155015962704

D2S441 Allele 11: 0.3365857988685797

D2S441 Allele 12: 0.0392109678533953

D2S441 Allele 13: 0.0304507799571702

D2S441 Allele 14: 0.2933445073629298

D2S441 Allele 15: 0.0430267358743375

D10S1248 Allele 12: 0.0293243925768293

D10S1248 Allele 13: 0.2741660081045789

D10S1248 Allele 14: 0.3126067817562505

D10S1248 Allele 15: 0.2060485379710324

D10S1248 Allele 16: 0.1377896948251211

D13S317 Allele 8: 0.1275029411425478

D13S317 Allele 10: 0.0613807787503547

D13S317 Allele 11: 0.3219161381478102

D13S317 Allele 12: 0.2755539874507353

D13S317 Allele 13: 0.0950999474237499

PentaE Allele 7: 0.1544247389507264

PentaE Allele 8: 0.0116863167850268

PentaE Allele 11: 0.1285457117137713

PentaE Allele 12: 0.2045067236058512

PentaE Allele 13: 0.1076800817235945

PentaE Allele 16: 0.0417360755222625

D16S539 Allele 5: 3.533128924904949e-4

D16S539 Allele 9: 0.112066944278855

D16S539 Allele 11: 0.3005228776649126

D16S539 Allele 12: 0.3039144616499847

D16S539 Allele 13: 0.1792679558649565

D18S51 Allele 12: 0.1335177890769616

D18S51 Allele 13: 0.1348591858198766

D18S51 Allele 14: 0.168505192798323

D18S51 Allele 15: 0.1434817061509933

D18S51 Allele 16: 0.134506505418883

D18S51 Allele 17: 0.1089219904335208

D18S51 Allele 18: 0.0713175995109202

D18S51 Allele 22: 0.0059372552581138

D2S1338 Allele 15: 5.655351447965016e-4

D2S1338 Allele 16: 0.0443238085006241

D2S1338 Allele 17: 0.2260707853748467

D2S1338 Allele 18: 0.0815070549093758

D2S1338 Allele 19: 0.1087933821516732

D2S1338 Allele 20: 0.1425131572873186

D2S1338 Allele 23: 0.1063202708800161

D2S1338 Allele 24: 0.1044126429126282

D2S1338 Allele 25: 0.1034214677907832

PentaD Allele 2.2: 0.0020868157561222

PentaD Allele 7: 0.0041736075522263

PentaD Allele 9: 0.208264255214379

PentaD Allele 10: 0.1022545842493137

PentaD Allele 11: 0.149

PentaD Allele 12: 0.2257919396258423

PentaD Allele 13: 0.2011690831116949

PentaD Allele 14: 0.0751252287850094

TH01 Allele 6: 0.2325467694894943

TH01 Allele 7: 0.1642863354025526

TH01 Allele 8: 0.1105848090833456

TH01 Allele 9: 0.1738965209542733

TH01 Allele 9.3: 0.3053981008454375

vWA Allele 14: 0.1041729331448433

vWA Allele 15: 0.1007074972382891

vWA Allele 16: 0.2074246851269154

vWA Allele 17: 0.275601161100602

vWA Allele 18: 0.2145693361130616

vWA Allele 19: 0.0796322798869906

D21S11 Allele 28: 0.1528397853963424

D21S11 Allele 29: 0.215870331449229

D21S11 Allele 30: 0.2398249361513519

D21S11 Allele 31: 0.0711561662823399

D21S11 Allele 31.2: 0.0920010869501008

D21S11 Allele 32: 0.013708391590555

D21S11 Allele 32.2: 0.0990671489445417

D7S820 Allele 7: 0.0197575302100237

D7S820 Allele 8: 0.1751856158478772

D7S820 Allele 9: 0.1601686611044745

D7S820 Allele 10: 0.2621182939056334

D7S820 Allele 11: 0.2038994850410368

D7S820 Allele 12: 0.1412019829889085

D7S820 Allele 13: 0.0321387616438468

D5S818 Allele 7: 0.0018440444680105

D5S818 Allele 10: 0.0663859924984179

D5S818 Allele 11: 0.3358868857219644

D5S818 Allele 12: 0.3383637096380166

D5S818 Allele 13: 0.1599062225180746

TPOX Allele 6: 0.0013171939872319

TPOX Allele 8: 0.5405645197383935

TPOX Allele 9: 0.1082728036027515

TPOX Allele 11: 0.2478931221312927

TPOX Allele 13: 0.0013171939872319

D8S1179 Allele 8: 0.0130724901988871

D8S1179 Allele 10: 0.0760315723893699

D8S1179 Allele 12: 0.1363781507427051

D8S1179 Allele 13: 0.3247614509143596

D8S1179 Allele 14: 0.2166471786107541

D8S1179 Allele 15: 0.1079722186490581

D12S391 Allele 18: 0.1854588903234353

D12S391 Allele 19: 0.1129026129015622

D12S391 Allele 19.1: 3.532562809066528e-4

D12S391 Allele 21: 0.1135385397122933

D12S391 Allele 22: 0.1069672847182726

D12S391 Allele 23: 0.0867600138312575

D12S391 Allele 25: 0.0133532767514195

D19S433 Allele 11: 0.0043103364137849

D19S433 Allele 12: 0.0855710231328339

D19S433 Allele 13: 0.2357265364781827

D19S433 Allele 14: 0.3486402156952063

D19S433 Allele 14.2: 0.0227530217773376

D19S433 Allele 15: 0.1614620698492373

D19S433 Allele 16: 0.1599906247253257

SE33 Allele 15: 0.1314153720079961

SE33 Allele 17: 0.0689898543265602

SE33 Allele 19: 0.0729527244179407

SE33 Allele 20: 0.0486692921666219

SE33 Allele 22: 0.0090428977656501

SE33 Allele 24.2: 0.0308880235690146

SE33 Allele 25.2: 0.0396257492042737

SE33 Allele 26.2: 0.0531394392142032

SE33 Allele 30.2: 0.051920130970559

D22S1045 Allele 11: 0.1345399568901373

D22S1045 Allele 15: 0.3623534186398688

D22S1045 Allele 16: 0.337268439080801

FGA Allele 20: 0.137637204272682

FGA Allele 21: 0.1793432463183378

FGA Allele 21.2: 0.0024034974516317

FGA Allele 23: 0.1485934049680537

FGA Allele 24: 0.1347367804276175

FGA Allele 27: 0.0039586613899145
